# Expression and localization of claudins-3 and -12 in transformed human brain endothelium

**DOI:** 10.1186/2045-8118-9-6

**Published:** 2012-02-29

**Authors:** Anja Schrade, Hadassah Sade, Pierre-Olivier Couraud, Ignacio A Romero, Babette B Weksler, Jens Niewoehner

**Affiliations:** 1 Faculty Pharmaceutical Biotechnology, Biberach University of Applied Sciences, Biberach an der Riss, Germany; 2 Pharma Research and Early Development (pRED), LMR Penzberg, Roche, Penzberg, Germany; 3 Institut Cochin, Department of Cell Biology, CNRS (UMR 8104), Paris, France; 4 INSERM, U567, Paris, France; 5 Department of Life Sciences, The Open University, Walton Hall, Milton Keynes, UK; 6 Division of Hematology-Medical Oncology, Weill Medical College of Cornell University, New York, NY, USA; 7 Biomedicum, University of Helsinki, Helsinki, Finland

**Keywords:** Blood Brain Barrier (BBB), hCMEC/D3, Claudins, Tight junction, Statins

## Abstract

**Background:**

The aim of this study was to characterize the hCMEC/D3 cell line, an *in vitro *model of the human Blood Brain Barrier (BBB) for the expression of brain endothelial specific claudins-3 and -12.

**Findings:**

hCMEC/D3 cells express claudins-3 and -12. Claudin-3 is distinctly localized to the TJ whereas claudin -12 is observed in the perinuclear region and completely absent from TJs. We show that the expression of both proteins is lost in cell passage numbers where the BBB properties are no longer fully conserved. Expression and localization of claudin-3 is not modulated by simvastatin shown to improve barrier function *in vitro *and also recommended for routine hCMEC/D3 culture.

**Conclusions:**

These results support conservation of claudin-3 and -12 expression in the hCMEC/D3 cell line and make claudin-3 a potential marker for BBB characteristics *in vitro*.

## Background

Members of the claudin superfamily are important protein constituents of tight junctions (TJs) in polarized epithelial and endothelial monolayers and play an important role in maintaining the paracellular barrier. At the blood brain barrier (BBB), claudins-1,-3, -5 and -12 have been shown to participate in the formation of TJs between brain microvascular endothelial cells *in vivo *[1-4]. Of these members, there has been a particular interest in claudin-5, as mice deficient for this protein showed size dependent transport restriction for molecules smaller than 800 Da. Claudin-12 is highly expressed at the TJs in murine development [3]. Claudin-3 expression in primary human, rat and murine brain endothelial TJs is no longer of debate since antibodies that are not cross-reactive to claudin-1 have been generated [5-7]. The loss of claudin-3 from the TJs in experimental allergic encephalomyelitis (EAE) demonstrates that it may be important for determining permeability in pathological conditions [4].

The hCMEC/D3 cell line is the best-characterized *in vitro *model system of the human blood brain barrier. The cells express endothelial specific and TJ markers including claudins-1 and -5 [8,9]. The aim of the present study was to establish the protein expression and localization pattern of the other brain endothelial specific claudins-3 and -12 in the hCMEC/D3 cell line.

## Methods

### Cell culture

Simvastatin (Sigma) was reconstituted as described [10]. hCMEC/D3 cells were cultured as described [9]. Primary endothelial cells (Cell Systems GmbH, Troisdorf, Germany) were cultured according to instructions. MCF7 human breast adenocarcinoma, MDCK-2 canine kidney, MEF1, Pea-13 and NIH-3T3 mouse fibroblast and U87 human glioblastoma cell lines were from American Type Culture Collection (LGC Standards GmbH, Wesel, Germany).

### Western blot analysis

Protein concentration of whole cell lysates generated in RIPA buffer were estimated (DC protein assay, Thermo Scientific, Bonn, Germany) and 20 μg of denatured lysates resolved on NuPAGE gels (Invitrogen, Darmstadt, Germany) were transferred onto nitrocellulose membranes (Invitrogen). Blocked membranes were incubated overnight with 1.5 μg/ml of Claudin-3 (Invitrogen) or 1 μg/ml claudin-12 (R&D Systems, Wiesbaden, Germany) antibodies. Membranes were incubated with HRP-conjugated IgG antibodies (Cell Signaling Technology, Frankfurt, Germany) for 1 h at RT and developed using Lumilight Plus detection reagents (Roche, Mannheim, Germany) and films exposed to the signal were developed with an X-Omat 1000 automatic processor (Kodak, Stuttgart, Germany).

### Immunocytochemistry

Coverslip cultures of hCMEC/D3 or MDCK cells were treated as described [9] and immunostained with 1.5 μg/ml of claudin-3 or 5 μg/ml claudin-12 antibodies followed by incubation with 10 μg/ml isotype-specific IgG conjugated to Alexa Flour^® ^488 (Invitrogen). Coverslips were mounted in UltraCruz mounting medium (Santa Cruz, Heidelberg, Germany) and images obtained with a Leica florescence microscope were processed using MetaMorph software (Molecular Devices, Biberach, Germany). Matched isotypes of the primary antibodies served as negative controls.

## Results

### Claudin-3 and -12 are expressed but only claudin-3 is localized at the TJs in the hCMEC/D3 cell line

**Figure 1:**
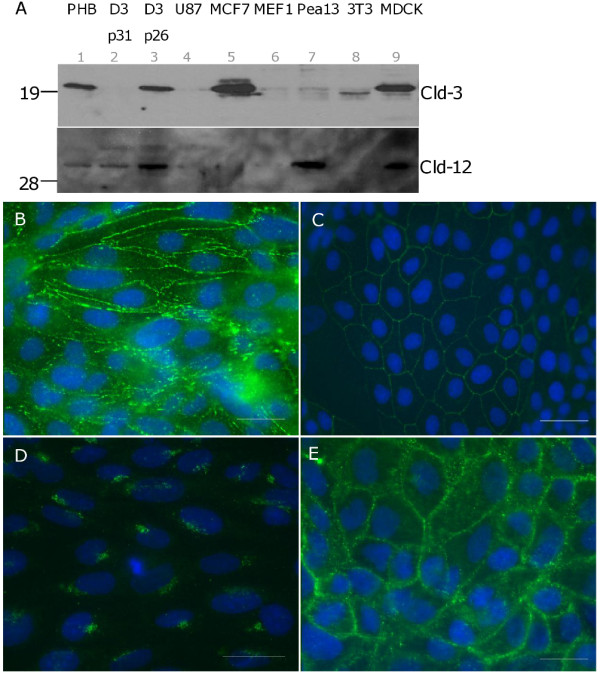
**Claudin-3 and Claudin-12 expression in hCMEC/D3 cells**. **A**: Immunoblots showing claudin-3 (Cld 3, upper panel) and claudin-12 (Cld 12, lower panel) expression in whole cell lysates derived from primary human brain (*lane 1*, PHB) and hCMEC/D3 endothelial cells (*lanes -2*, D3 p31 and -*3*, D3 p26) in addition to glioblastoma cells (*lane 4*, U87) or cells of fibroblast origin (*lanes-6*, MEF1; -*7*, Pea-13; -*8*, NIH 3T3), epithelial (*lanes -5*, MCF-7 and -*9*, MDCK) origin. B-E: hCMEC/D3 cells (**B**, **D**) grown to confluence and further rested for 48 h or confluent cultures of MDCK cells (**C**, **E**) were stained with antibodies to claudin-3 (**B**, **C**) or claudin-12 (**D**, **E**). Isotype matched antibodies did not stain cells (data not shown). Scale bar = 25 μm.

We compared the expression of claudin-3 and claudin-12 in hCMEC/D3 cells to primary human brain endothelium and other non-endothelial based cell lines. Under reducing conditions (Figure 1A), a ~23 kDa protein band corresponding to the size of claudin-3 was observed in endothelial and epithelial cells (lanes, 1, 2, 3, 5, and 9). Claudin-3 levels are comparable between primary brain endothelium (PHB, lane 1) and transformed (hCMEC/D3, lane 3). Claudin-3 is strongly expressed in epithelium where protein levels in MCF7 (lane 5) and MDCK cells (lane 9) are higher than that observed in the brain endothelial cells (lanes 1, 3). Interestingly, we see a loss of the claudin-3 protein in hCMEC/D3 cells from passage 31 (lane 2) compared to cells derived from passage 26 (lane 3).

Claudin-12 is a 27 kDa protein. Like other claudins, it is regulated by post-translational modifications, a possible reason for the higher molecular weight band observed. It is expressed in primary and transformed brain endothelium (Figure 1A, lanes 1, 2 and 3). The decrease in protein levels with increase in passage number also appears to be consistent for the claudin-12 protein (lanes 2 and 3). Claudin-12 is present in MDCK (lane 9) cells. Surprisingly it is seen in the Pea-13 (lane 8) cells even though it is absent from its parent line, MEF-1 (lane 7).

We proceeded to check the localization of the expressed claudin-3 and claudin-12 proteins in the hCMEC/D3 cell line (Figure 1B-E). Claudin-3 shows a distinct localization at the tight junctions (Figure 1B) also seen in the MDCK cells (Figure 1C). However, we did not detect claudin-12 at the TJ in the hCMEC/D3 cells but observed immunoreactivity reminiscent of a protein associated with the ER network (Figure 1D). We checked if the antibody can detect protein at the TJ and indeed the protein is observed at cell-cell borders in the MDCK cells using this antibody (Figure 1E).

### No modulation of claudin-3 protein expression and its localization by simvastatin

**Figure 2:**
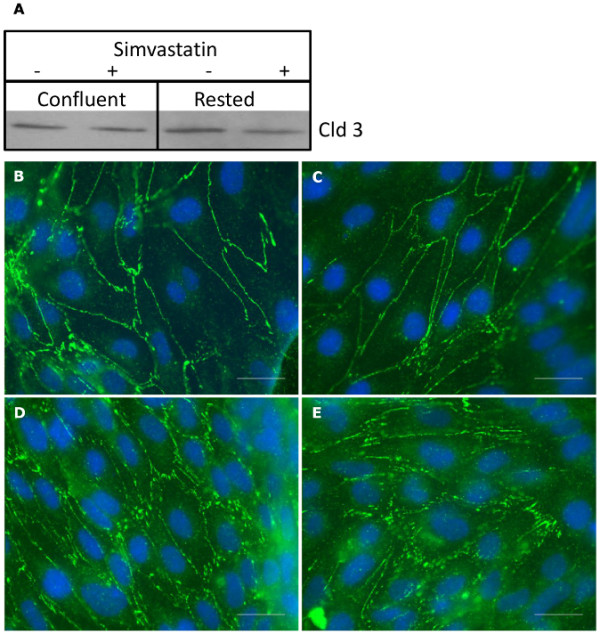
**Influence of simvastatin on hCMEC/D3 confluent and rested monolayers**. hCMEC/D3 cells were grown to confluence or further rested for 48 h in medium lacking growth factors. In addition, confluent or rested cultures were incubated with 1 nM Simvastatin overnight at 37°C. **A: **Immunoblot showing claudin-3 expression under indicated conditions. **B**-**E: **Immunolocalization of claudin-3 in confluent (**B**,**C**) or rested (**D**,**E**) cells in the presence (**B**,**D**) or absence (**C**,**E**) of simvastatin. Cells were immunostained with an antibody to the claudin-3 protein and visualized with goat anti-rabbit IgG conjugated to Alexa Fluor^® ^488. Scale bar = 25 μm.

Simvastatin, a member of the statin family, has been proposed to positively modulate the poor TEER and increased paracellular permeability inherent to the hCMEC/D3 culture [11]. We investigated if simvastatin improves the barrier properties by up-regulating expression or localization of claudin-3. Confluent or rested hCMEC/D3 cells were cultured with or without 1 nM simvastatin for 12 h (Figure 2). Western blot analysis (Figure 2A) shows constant levels of claudin-3 protein between the different conditions. While simvastatin treatment does not appear to change claudin-3 localization, rested cultures show a slightly more irregular staining pattern. (Figure 2B, C).

## Discussion

The aim of this study was to characterize claudins-3 and -12 inhCMEC/D3 cells. Our data support the following conclusions:

1. Expression of claudins-3 and -12, but only claudin-3 is localized to the tight junction (Figures 1 and 2).

2. Common feature of claudins-3 and -12 is their loss in higher passage numbers (Figure 1).

3. Simvastatin improvement of the monolayer integrity is not mediated via increase in the expression or localization of claudin-3.

We detected claudin-12 protein in primary and transformed brain endothelium, but not at the TJ. In murine development, claudin-12 is predominantly seen at the TJs [4], while data on adult brain are only available at the mRNA level: findings by Belanger et al. support claudin-12 as a marker for metabolic disturbances affecting brain function in the adult [12]. In an immortalized murine cell culture model, only claudin-12 mRNA levels were down-regulated under conditions mimicking hyperammonemia. Taken together, it cannot be excluded that claudin-12 exists as an intracellular protein in the adult brain serving an as yet unknown function, or its localization to the TJ *in vitro *might require exquisite signaling input from other members of the neurovascular unit like pericytes and astrocytes. We did not test the hCMEC/D3 cells in co-culture with astrocytes as they are not responsive to glial cell conditioning in our experimental set-up.

So far, data on claudin-3 expression in immortalized human brain endothelium is supported by data from iHBMEC, an immortalized human brain endothelial cell line [13]. The loss of the protein from tight junctions in experimental EAE demonstrates its importance for determining permeability in pathological conditions [4]. In hCMEC/D3 cells, claudin-3 is observed as a combination of continuous lines and a serrated pattern at the tight junctions, depending on culture conditions. The protein is not detected in cells that no longer exhibit the full complement of BBB properties in culture: TEER values of hCMEC/D3 cells at early passages are in the range of 35-40 ohm/cm^2^, while in p31 cells, the TEER falls to 20-22 ohm/cm^2^. This supports the finding of Wolburg et al. whose data identify claudin-3 as the primary marker for detecting loss in BBB integrity *in vivo *[4]. Ifergan et al. observed that simvastatin improved barrier properties of brain endothelial monolayers without affecting expression or localization of occludin, ZO-1, -2, VE-cadherin and JAM-1 proteins [10]. Claudin-3 protein expression or localization is also not modified by addition of simvastatin to the culture medium. Claudins-1 and-5 remain to be investigated for the effects of simvastatin.

In conclusion, we present data confirming the expression of claudins-3 and-12 in the hCMEC/D3 cell line. Claudin-3 is a possible marker for hCMEC/D3 monolayer integrity in studies investigating modulation of BBB *in vitro *under pathological conditions. Further work to identify the function of claudin-12 in the adult brain and its binding partners remains to be investigated.

## Abbreviations

BBB: Blood brain barrier; hCMEC/D3: Human cerebral microvascular endothelial cell D3; TJ: Tight junction; ZO-1, 2: Zona occludens-1, 2; JAM-1: Junctional adhesion molecule-1; VE-Cadherin: Vascular endothelial-Cadherin; iHBMEC: Immortalized human brain endothelial cell line; RNA: Ribonucleic acid.

## Competing interests

Anja Schrade, Hadassah Sade and Jens Niewoehner are employees of Roche Diagnostics GmbH.

## Authors' contributions

HS, JN: conceived and designed the study; AS, HS: performed the experiments; AS, HS and JN: analyzed the data; HS, JN: wrote the paper. All authors read and approved the final manuscript.
